# Case Report: Total atherosclerotic occlusion of perforators in anterolateral thigh flap

**DOI:** 10.3389/fsurg.2026.1732446

**Published:** 2026-02-05

**Authors:** Seok Woo Lee, Hyonsurk Kim

**Affiliations:** 1 Department of Plastic & Reconstructive Surgery, Dankook University Hospital, Cheonan, Republic of Korea; 2 Department of Plastic & Reconstructive Surgery, Dankook University College of Medicine, Cheonan, Republic of Korea

**Keywords:** anterolateral thigh flap, atherosclerosis, case report, perforator, vessel occlusion

## Abstract

Atherosclerosis and calcification of vessels, commonly encountered in diabetic patients, is a well-known complicating factor in microsurgical reconstruction. However most studies on this problem have focused on axial vessels and their major branches. We present a case of total atherosclerotic occlusion of perforators rising from intact lateral circumflex femoral artery branches in bilateral anterolateral thigh (ALT) flap elevation. A 51-year-old male with a history of diabetes mellitus, ischemic heart disease and kidney transplantation presented with necrotizing fasciitis of the left foot. Serial debridement and antibiotics stabilized the patient, who was thereafter referred for foot reconstruction. Ipsilateral ALT free flap reconstruction was planned, however although preoperative computed tomography angiography (CTA) confirmed patency of the left lower extremity arterial system and Doppler mapping enabled uneventful suprafascial flap elevation based on a large pulsatile perforating vessel, the perforator was severely calcified on visual inspection, and no flap perfusion was found after perforator isolation. The same findings including total occlusion of the perforator pedicle on cross-section were discovered on contralateral ALT flap elevation. Foot reconstruction was ultimately achieved using a thoracodorsal artery perforator flap, which displayed no atherosclerotic lesions. Even with nonspecific lower extremity CTA and Doppler findings and clinically patent axial arteries, perforators perfusing major flaps can be totally occluded in atherosclerotic patients. More sensitive diagnostic tools such as color duplex sonography or high-quality CTA should be employed for preoperative perforator mapping in atherosclerosis-risk patients.

## 1 Introduction

The lower leg and foot regions present unique challenges in soft tissue reconstruction due to the innate thinness of subcutaneous tissue and paucity of recruitable locoregional flap options. Free tissue transfer procedures are frequently required in this area for durable coverage and limb salvage. The anterolateral thigh (ALT) flap is a time-tested workhorse free flap owing to its long vascular pedicle, broad skin paddle, and minimal donor site morbidity (1). Though it does have variations in perforator position and course, preoperative perforator mapping with Doppler ultrasonography and computed tomographic angiography (CTA) has improved planning and shortened dissection. Nevertheless, each of these modalities does have limitations in availability, perforator-level resolution, and operator dependence (1–4). Such imaging studies can still struggle with very small-caliber perforators and also with characterizing intraluminal atherosclerotic disease and calcification—well-known complicating factors in microsurgical reconstruction (5–7).The clinical issues caused by such factors have been intensively studied, nevertheless most investigations have focused on named axial arteries and their major branches, with relatively less attention toward perforator-level pathology that can directly determine flap viability (8, 9).

We report a rare case of bilateral total atherosclerotic occlusion of ALT perforators arising from intact lateral circumflex femoral artery (LCFA) branches in a high-risk patient. Despite nonspecific preoperative CTA and handheld Doppler findings, both ipsilateral and contralateral ALT flaps displayed no perfusion after elevation based on isolated perforators. Definitive coverage was achieved with a thoracodorsal artery perforator (TDAP) free flap. This case highlights the potential gap between macrovascular patency and true perforator patency and provides practical considerations for imaging and flap selection in atherosclerosis-risk patients.

## 2 Case description

**Figure 1:**
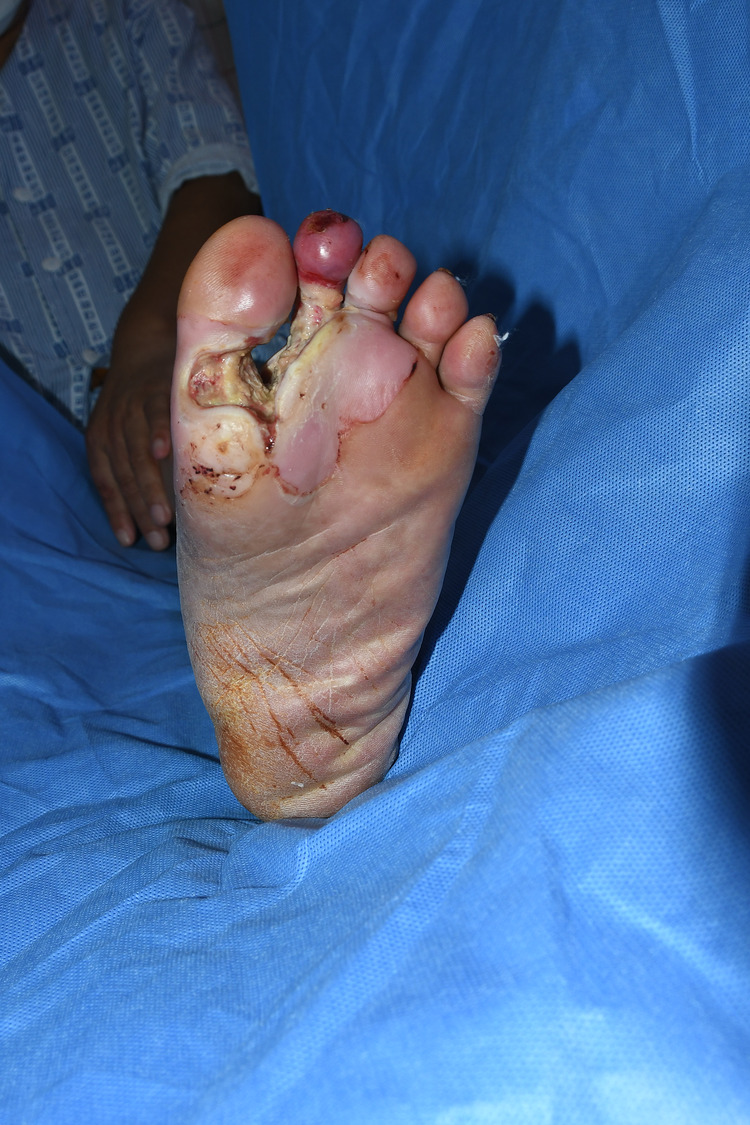
Initial state of foot when referred for reconstruction.

A 51-year-old man with a history of diabetes mellitus, ischemic heart disease, and previous kidney transplantation presented with fever, chills, and painful swelling of the left foot. The patient was initially evaluated and hospitalized by our Orthopedic Surgery department with a diagnosis of necrotizing fasciitis. After serial surgical debridement and intravenous antibiotics stabilized his general status, the patient was referred to our department for reconstructive coverage of the foot (Figure 1). After additional surgical debridement including amputation of the non-viable second toe which displayed decreased circulation, negative pressure wound therapy was applied while a reconstructive plan was established.

**Figure 2:**
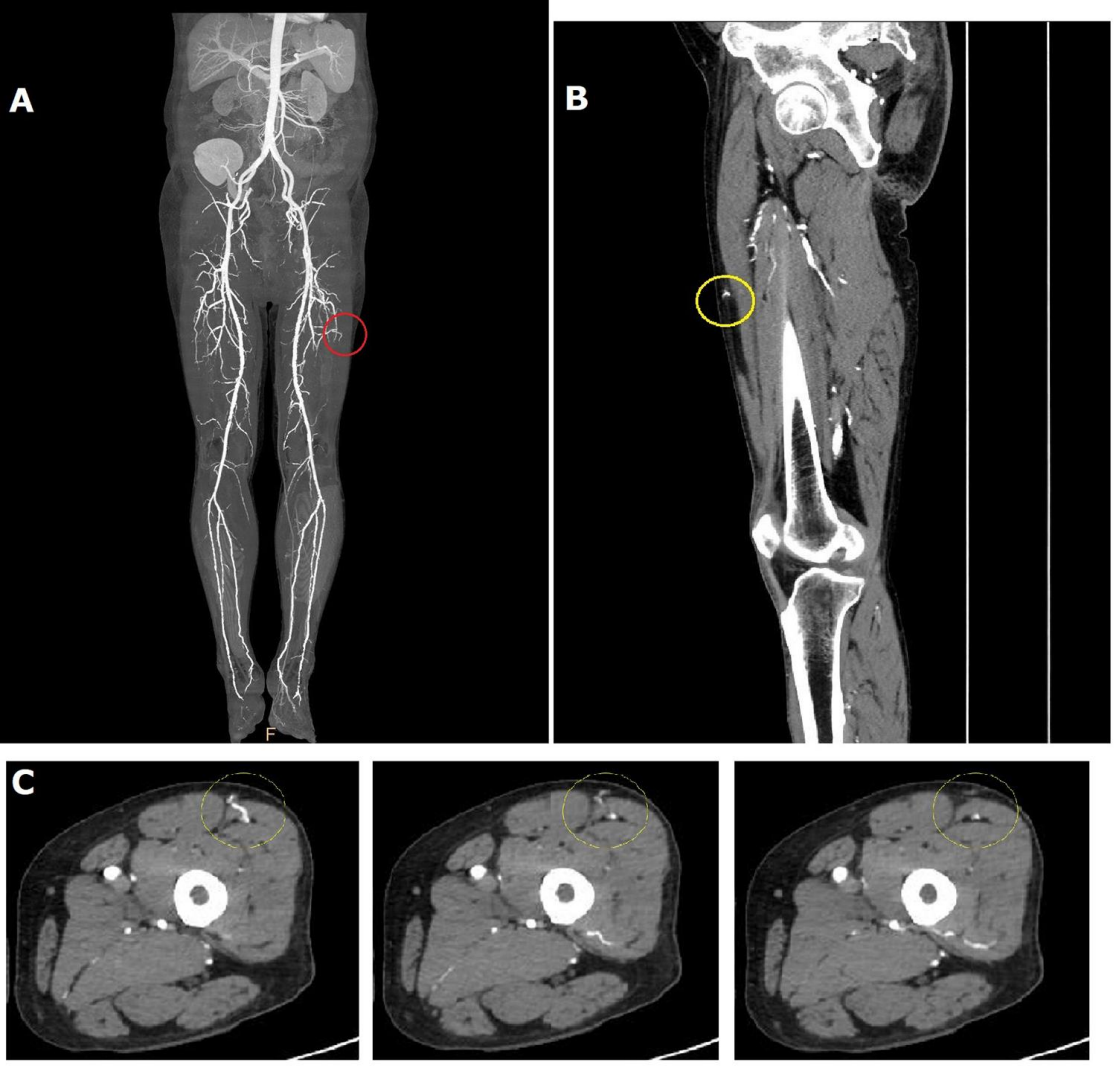
Preoperative computed tomographic angiography (CTA) findings. Contrast timing was set to start scanning when the iliac artery displayed intense enhancement, generally between 35 and 40 s. Though severely atherosclerotic with diffuse calcific plaques, grossly intact major vasculature patency was confirmed in the left lower limb donor and recipient sites **(A)** The flap was elevated based on a perforator with a robust audible Doppler signal in the customary circular area for anterolateral thigh flap dissection which corresponded to a musculocutaneous perforator visible on CTA [**(B)**, sagittal view; **(C)**, axial view], indicated by yellow circles. The area of the flap perforator is also depicted in a red circle in **A**.

Based on preoperative lower extremity vasculature patency assessment with 128 channel multidetector-row CTA and handheld acoustic Doppler probing, an ipsilateral ALT free flap was chosen for defect coverage. Though severe atherosclerosis with diffuse calcific plaques was visible, CTA findings (slice thickness: axial 2.5 mm, coronal and sagittal 1 mm) confirmed gross patency of the left lower extremity major arterial system (Figure 2A), and Doppler mapping enabled uneventful suprafascial flap elevation based on a large pulsatile perforating vessel located at the typical circular site about halfway between the anterior superior iliac spine and superolateral patella border, which corresponded to a musculocutaneous perforator visible on CTA (Figures 2B,C).

**Figure 3:**
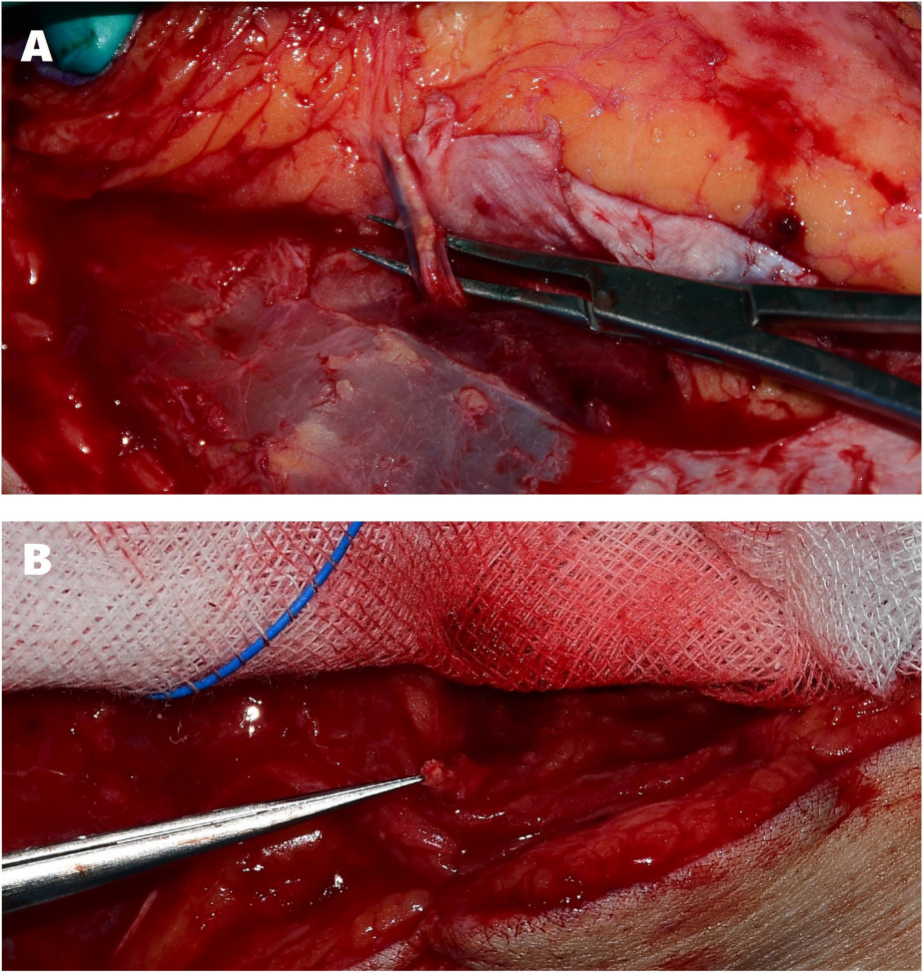
Intraoperative perforator findings. **(A)** Pulsatile but severely calcified left anterolateral thigh perforator. **(B)** Cross section of contralateral thigh perforator displaying near-total stenosis with no blood flow.

**Figure 4:**
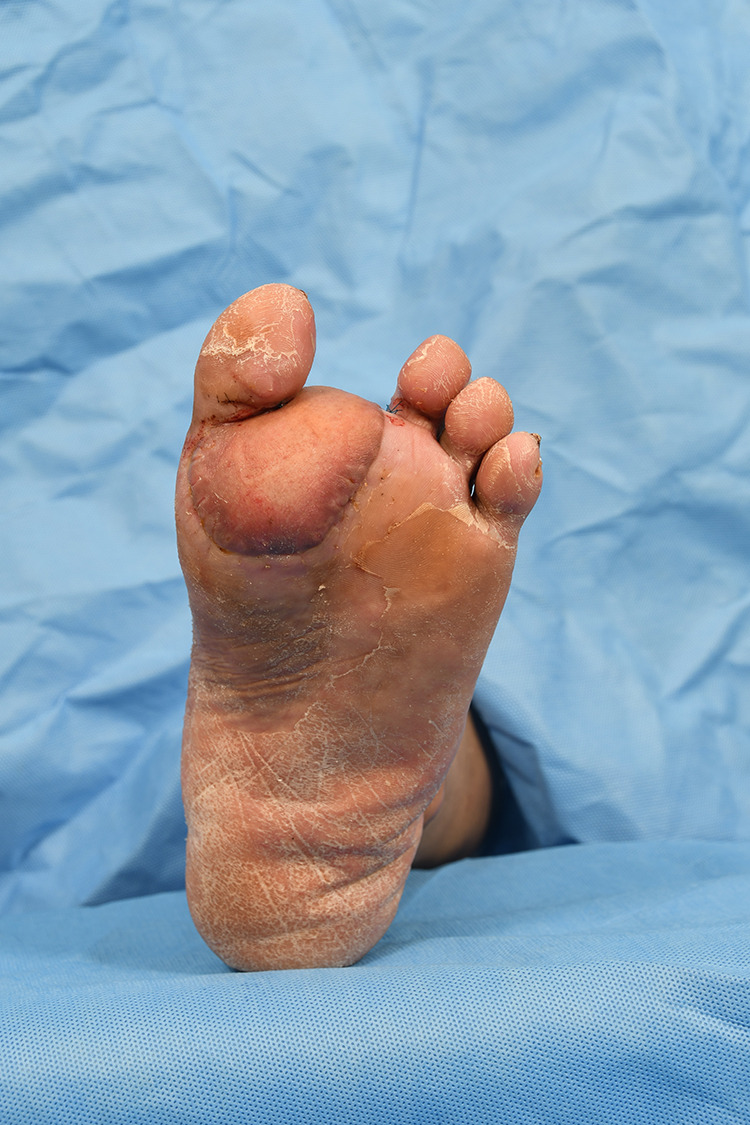
Successful reconstruction using a thoracodorsal artery perforator flap. 2-month postoperative state.

However the perforator displayed severe calcification on visual inspection, and after fully elevating the flap with isolation of the perforator no perfusion was observed in the skin paddle. The perforator pedicle was sectioned and demonstrated severe atherosclerosis with no flow and near-total luminal occlusion (Figure 3A). Another ALT flap was elevated from the contralateral thigh, again based on a well-audible perforator found by handheld acoustic Doppler probing in the same customary circular area. Again, the same findings of severe calcification with near-total stenosis of the perforator pedicle were discovered (Figure 3B), yielding another ischemic flap with no visible perfusion after division. Foot coverage and salvage was ultimately achieved using a TDAP flap which displayed no such occluding atherosclerotic lesions. The 10 cm-length vascular pedicle of the flap was anastomosed to the dorsalis pedis artery at the metatarsal base level, which had been dissected and evaluated for normal blood flow before initiation of ALT flap elevation. Due to concerns for postoperative edema and pedicle compression, the anastomosis site was not immediately closed, and instead a collagen dressing was applied over the exposed vessels. The skin was closed 2 weeks later, after verification of TDAP flap survival and resolution of foot edema. The postoperative course was uneventful, with complete flap survival and successful wound healing for stable foot salvage (Figure 4).

## 3 Discussion

This case demonstrates that even with patent axial arterial inflow and apparently favorable preoperative Doppler signals, perforators crucial to flap perfusion can be selectively affected by clinically significant atherosclerosis. Perforators of ALT flaps on both sides were grossly calcified and occluded on cross-section, leading to immediate flap ischemia after elevation, regardless of intact LCFA branches on preoperative CTA and strong Doppler signals (1, 10). This example emphasizes that macrovascular patency does not guarantee perforator patency—which is the level that actually determines viability in such perforator flaps— especially in patients with comorbidities such as diabetes, renal disease, and advanced atherosclerosis (8, 9).

Handheld acoustic Doppler ultrasonography, easy to use and sensitive to flow, is perhaps the most widely accessible preoperative diagnostic tool for microsurgery, but limited by poor specificity. Good acoustic signals can represent signal transmission from deeper axial or septal vessels rather than an actual cutaneous perforator, or calcification can produce acoustic shadowing that obscures a short occluded segment with intact flow proximal to the lesion. Perforator mapping work has demonstrated discordance between preoperative Doppler marking and intraoperative perforator entry points (2, 4, 11, 12).

On the other hand, color duplex ultrasonography can define course, caliber, and waveforms for targeting and selecting the largest, best-located perforators, with successful use in guiding freestyle perforator flap reconstruction including in the ALT region (3, 13). However the results hinge on the expertise of the user, including selection of probe frequency and protocol. Duplex examination also requires more time than simple handheld Doppler and is not universally available on short notice. Though these ultrasound devices are equipped in the Plastic and Reconstructive Surgery departments of certain institutes, our department does not yet possess such imaging devices, and the selective departments of our institute that are equipped with these high-quality ultrasound devices were not familiar with perforator mapping. Multidetector-row CTA can improve operative efficiency, especially when high resolution and thin-slice acquisition is coupled with surgeon-directed processing, however standard lower extremity protocols may fail to consistently resolve small perforators or fail to detect short segments of diseased vessels, as in this case. Radiation and iodinated contrast risks should also be considered in susceptible patients (1, 5, 10). High-resolution magnetic resonance angiography can demonstrate superb vessel conspicuity without the risk of radiation, but is also limited by accessibility and cost in many institutes, as well as the choice of contrast material (14). In high-risk patients, our experience with this case suggests upgraded preoperative mapping where available; thin-slice CTA centered on perforator course combined with targeted duplex assessment to better differentiate calcified or occluded perforators from reliable pedicles (7).

Pathophysiologically, the LCFA system can be affected in atherosclerotic patients, especially the descending branch. Comparative studies have demonstrated various degrees of stenosis of these vessels in atherosclerosis-risk patients, with smoking, hypertension and diabetes being among factors cumulating risk burden (6, 8). However these studies only focused on major axial vessels and did not evaluate smaller perforators. When the superficial femoral artery is occluded, collateral circulation through the deep femoral artery and descending branch of the LCFA become crucial to distal perfusion, but even these collateral adaptations do not fully preclude superimposed disease at the level of perforators that determine ALT flap viability (9).

When faced with a calcified and stenotic perforator while elevating perforator flaps, reconstructive surgeons should first recognize that preoperative evaluation such as Doppler ultrasound or CTA can produce false-positive findings. Standard intraoperative measures to ease vasoconstriction such as lidocaine or warm saline irrigation and adventitial stripping can be employed if utilizing a different patent perforator is unfeasible. If perfusion is inadequate despite anatomically correct flap elevation, alternative donor sites with pedicles less prone to calcific occlusion should be considered. As vessels in the trunk or upper extremity are less prone to atherosclerotic stenosis and occlusion, flaps based on these sites (such as the TDAP or radial forearm flap) could provide healthier long pedicles to reach even proximal recipient vessels, avoiding heavily calcified distal branches (7, 15). In high-risk patients, if upgraded preoperative perforator mapping such as color duplex ultrasound is unattainable then these flaps should be considered as primary options instead of lower extremity-based flaps such as the ALT flap.

This report of a single case has inherent limitations but still stands as an example that perforator-level atherosclerosis can be an isolated cause of ALT non-perfusion regardless of intact inflow from axial branches. Perforator patency, especially in the lower extremity, should be approached as an independent question in high-risk patients. Acknowledge the potential shortcomings of acoustic Doppler probing, optimize imaging studies that specifically target perforators, and be ready to utilize a back-up donor such as the TDAP flap whenever perforator patency is in question with a low threshold to intraoperatively change surgical strategy. Future studies involving larger cohorts of comorbid patients could help in further defining the incidence and clinical impact of perforator-level atherosclerosis.

## Data Availability

The original contributions presented in the study are included in the article/Supplementary Material, further inquiries can be directed to the corresponding author.
